# Linear versus conformational epitopes of three cow's milk allergens

**DOI:** 10.1186/2045-7022-4-S2-P23

**Published:** 2014-03-17

**Authors:** Katrine Lindholm Bogh, Jeanette Lund Madsen, Stine Kroghsbo, Charlotte Bernhard Madsen, Vibeke Barkholt

**Affiliations:** 1 Technical University of Denmark, National Food Institute, Søborg, Denmark; 2 Technical University of Denmark, Department of Systems Biology, Kgs. Lyngby, Denmark

## Background

Characterisation of epitopes, including relationship between linear and conformational epitopes, is an essential task for understanding the basic molecular mechanisms of food allergy.

The aim of this study was to investigate the importance of linear versus conformational antibody epitopes of the three cow's milk allergens; β-lactoglobulin (BLG), α-lactalbumin (ALA) and β-casein.

## Method

Specific antibodies were raised in a Brown Norway (BN) rat model of food allergy. BN rats were dosed either (1) intraperitoneally (i.p.) with the purified native cow's milk allergens with or without the use of Al(HO)3 as adjuvant or (2) orally with skimmed milk powder without the use of adjuvant. The specific IgG1 and IgE antibody responses were analysed against native and denatured allergens by means of different ELISAs.

## Results

A decrease in antibody reactivity towards the denatured allergen compared to the native allergen was seen for all three cow's milk proteins, independent of the administration route. This shows an importance of conformational epitopes for all three allergens. The specific antibody response towards denatured BLG was statistically significantly lower than towards native BLG. The BLG results showed that about 15 times more antibodies were raised against conformational epitopes compared to linear epitopes and that this was independent of the administration route. In contrast, the specific antibody response towards denatured β-casein was shown not to be statistically different from that of the native protein, again independent of the administration route. Here only around half of the antibodies seemed to be raised against conformational epitopes. Whereas both BLG and β-casein showed no differences between administration routes, ALA showed a statistically significant difference between the denatured and native allergen state, only with the use of i.p. administration. Here more than 50 times more antibodies had been raised against conformational epitopes compared to linear epitopes. In contrast, this number was only 4 times when dosing via the oral route. Moreover, the study showed that use of adjuvant strongly influenced the importance of conformational epitopes, as Al(OH)3 increased the reactivity towards linear epitopes.

## Conclusion

Collectively the study shows that the role of conformational epitopes greatly depends on the individual allergen, the use of adjuvant, and may for some be affected by their structural stability.

